# Fifteen Years of Annual Mass Treatment of Onchocerciasis with Ivermectin Have Not Interrupted Transmission in the West Region of Cameroon

**DOI:** 10.1155/2013/420928

**Published:** 2013-04-17

**Authors:** Moses N. Katabarwa, Albert Eyamba, Philippe Nwane, Peter Enyong, Joseph Kamgno, Thomas Kueté, Souleymanou Yaya, Rosalie Aboutou, Léonard Mukenge, Claude Kafando, Coulibaly Siaka, Salifou Mkpouwoueiko, Demanga Ngangue, Benjamin Didier Biholong, Gervais Ondobo Andze

**Affiliations:** ^1^The Carter Center, Atlanta, GA, USA; ^2^The Carter Center, Yaoundé, Cameroon; ^3^Research Foundation for Tropical Diseases and Environment, Buea, Cameroon; ^4^Filariasis Research Centre, Yaoundé, Cameroon; ^5^Faculty of Medicine and Pharmaceutical Sciences, University of Douala, Douala, Cameroon; ^6^Ministry of Public Health, North Region, Garoua, Cameroon; ^7^Ministry of Public Health, Yaoundé, Cameroon; ^8^African Programme for Onchocerciasis Control, Ouagadougou, Burkina Faso; ^9^Ministry of Public Health, West Region, Bafoussam, Cameroon

## Abstract

We followed up the 1996 baseline parasitological and entomological studies on onchocerciasis transmission in eleven health districts in West Region, Cameroon. Annual mass ivermectin treatment had been provided for 15 years. Follow-up assessments which took place in 2005, 2006, and 2011 consisted of skin snips for microfilariae (mf) and palpation examinations for nodules. Follow-up *Simulium* vector dissections for larval infection rates were done from 2011 to 2012. mf prevalence in adults dropped from 68.7% to 11.4%, and nodule prevalence dropped from 65.9% to 12.1%. The decrease of mf prevalence in children from 29.2% to 8.9% was evidence that transmission was still continuing. mf rates in the follow-up assessments among adults and in children levelled out after a sharp reduction from baseline levels. Only three health districts out of 11 were close to interruption of transmission. Evidence of continuing transmission was also observed in two out of three fly collection sites that had infective rates of 0.19% and 0.18% and ATP of 70 (Foumbot) and 300 (Massangam), respectively. Therefore, halting of annual mass treatment with ivermectin cannot be done after 15 years as it might escalate the risk of transmission recrudescence.

## 1. Introduction

Onchocerciasis, a leading cause of blindness, is due to human infection with *Onchocerca volvulus*, a parasitic worm that forms nodules under the skin. The female worms produce microfilariae (mf) that live in the nodules, inflame the skin, and enter the eyes, giving rise to inflammatory lesions. The mf which are picked up by *Simulium* flies during a blood meal develop into L1, L2, and L3 larval stages. The L3 (infective) larvae may be passed on to others on subsequent bites, thus completing the life cycle. Black flies breed in fast flowing rivers and streams, lending the name “river blindness” to the condition. Ivermectin is a safe and effective microfilaricidal drug that has been donated by Merck & Co (Mectizan) since 1987 for mass treatment of onchocerciasis. This medicine kills the microfilaria and reduces the risk of developing eye and skin diseases associated with the infection. Ivermectin also reduces the fecundity of adult worms and apparently shortens their life span [1]. However, treatment may be given for undetermined period of time in order to effect cure [2, 3].

Onchocerciasis control in Africa has been very successful over the last two decades. In West Africa, the Onchocerciasis Control Programme (OCP) eliminated onchocerciasis as a public health problem from the savanna areas of 11 countries through vector control and ivermectin treatment before its closure in 2002. However, surveillance and mass treatment with ivermectin activities are still going on [4, 5]. Outside OCP areas, control of onchocerciasis in Africa is the responsibility of the African Programme for Onchocerciasis Control (APOC), a partnership of the World Bank, World Health Organisation, a coalition of Non-Governmental Development Organisations, and affected African countries which was established in December 1995. Currently, over 68 million people are being treated with a single annual dose of ivermectin every year in Africa [6, 7].

The goal of APOC was to establish a mechanism for sustained delivery of an annual dose of ivermectin, thereby achieving reduction of prevalence of onchocerciasis to a point where the disease was no longer of public health or socioeconomic concern. However, the APOC goal of eliminating onchocerciasis as a public health problem (EPHP) was not quantitatively defined, but logically taken to be when prevalence is driven below the original baseline threshold required to launch the mass ivermectin treatment program, which is an onchocercal nodule rate ≥20% or an mf rate ≥40% [8]. Yet achieving EPHP as defined at these levels did not necessarily indicate interruption of transmission. In case transmission is not interrupted, halting mass treatment may result into disease recrudescence [8, 9].

Onchocerciasis control in West Region began in 1996 with Carter Center and Lions Clubs International Foundation (LCIF) assistance through a single annual dose of ivermectin as recommended by World Health Organisation. Later in 1998, the region began receiving additional funds from APOC for a period of five years for the implementation of community directed treatment with ivermectin (CDTI) for onchocerciasis control that was later to be sustained by the government of Cameroon. The five years of funding were followed by support for advocacy and replacement of capital equipment for three more years. The government of Cameroon did not bridge the financial gap and take over the project as expected [10]. Therefore Carter Center continued its technical and financial assistance to the CDTI activities to the West Region. APOC continued to provide limited funds for specific activities, but only when they were deemed critical for sustainability of CDTI activities.

The recent study in hyperendemic foci in Mali and Senegal showed that 15 to 17 years of annual ivermectin treatment had eliminated onchocerciasis transmission and that mass treatment could be safely stopped [11]. This provided evidence that in some areas, an annual dose of ivermectin could eliminate onchocerciasis. The two criteria used by Diawara et al. to make this determination were entomological (<0.5 infected flies/1000) and epidemiological (<5% mf prevalence in all communities examined and <1% in 90% communities examined). However, in the south of the Rio Falema focus, there were seven villages with mf prevalence between 1% and 13% after 15–17 years of annual treatment that were not discussed in the paper. This important report called for additional studies in areas with similar durations of treatment to determine if other successes could be documented across various onchocerciasis ecological transmission zones in Africa. In response to this report, an impact study conducted in North Region of Cameroon showed substantial reduction in mf and nodule prevalences in three health districts that had received up to 17 years of annual mass treatment with ivermectin [8]. Results of this study revealed that, while the onchocerciasis foci in the health districts of Tchollire and Rey Bouba appeared to fulfill the Diawara criteria of elimination, in the Health District of Touboro, transmission was still going on. The objective of the present study was to determine whether 15 years of annual treatment with ivermectin in West Region, a different ecological setting, had interrupted the transmission of onchocerciasis as had been observed in Mali and Senegal [11].

## 2. Materials and Methods

### 2.1. Study Area

**Figure 1:**
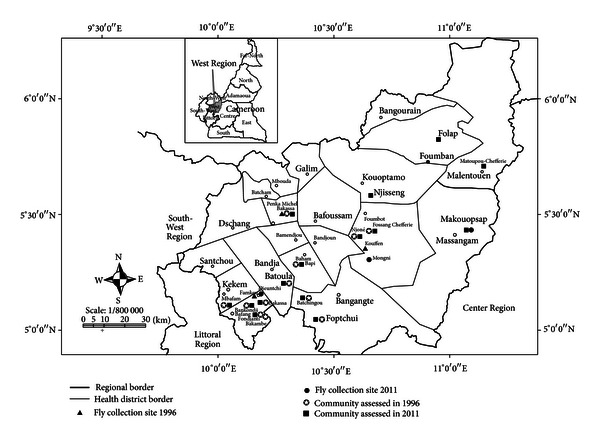
Map of West Region of Cameroon showing the study areas.

West Region is close to 14,000 km² of territory located in the central-western portion of the Republic of Cameroon (Figure 1). It borders the Northwest Region to the northwest, the Adamawa Region to the northeast, the Centre Region to the east and southeast, the Littoral Region to the southwest, and the Southwest Region to the west. The West Region is the smallest of Cameroon's ten regions in area, yet it has the highest population density with a total population of about 1,699,000 people living in 20 health districts. The mountainous terrain creates many perennial fast-running rivers that support breeding of black flies which transmit onchocerciasis throughout the year. The vegetation consists of thick forest in the western and eastern parts of the region while the middle part is a transition from forest to savannah woodland. The main vector of onchocerciasis in West Region of Cameroon is *Simulium squamosum*, a member of *Simulium damnosum* complex.

### 2.2. History of Mass Treatment with Ivermectin

Annual mass treatment with ivermectin commenced in 1996 and was carried out annually through 2010 in all the 20 health districts. Validation of treatment coverage through household face-to-face interviews was conducted every year from 2003 to 2010 in order to ensure that what was reported was correct. This also presented opportunities to identify issues that could be improved upon in order to attain and sustain the desired treatment coverage of at least 90% of the ultimate treatment goal (UTG). UTG is the sum of all eligible persons for treatment (minus children <5 years) among the total number of people at risk living in all at-risk communities in the onchocerciasis endemic area that the program ultimately has to treat [12]. The individuals in the samples selected for interviews were obtained through multistage random sampling in a homogenous population at 95% confidence level where a ±5% sampling error was accepted [13]. The data was entered and analysed in (Epi Info Version 6.04; CDC, Atlanta, GA, USA). Since every district and community had equal chances of being selected every year, the results obtained were considered representative of the annual treatment coverage [14–16]. Annual validation of treatment coverage reports showed achievement of at least 90% of UTG every year (Table 1). Therefore the surveyed treatment coverage validated the reported treatment coverage from 2003 to 2010.

### 2.3. Baseline Assessments 1996

Before commencement of mass treatment with ivermectin, baseline entomological and parasitological surveys were conducted in this region in 1996 with Carter Center support.

#### 2.3.1. Baseline Parasitological Assessments, 1996: Microfilaria (mf) survey

Baseline data were secured from 12 communities belonging to 7 health districts: Bafang (3), Baham (1), Banja (1), Bangangte (2), Foumbot (2), Kekem (1), and Penka-Michel (1). After obtaining consent, an individual's name, age, and gender were recorded on a registration form. Adults of 20 years and above who had lived in their respective communities for at least 10 years were selected for skin snipping. A total of 931 adults from these communities were assessed for mf. Also, 185 resident children (102 from one community in Bafang and 83 from two in Foumbot) were assessed. It was not possible to skin snip children in other baseline communities. Two skin snips were taken, one from the posterior iliac crest and another from the buttock, using a corneoscleral punch. The skin snips were placed immediately in wells of microtitration plates containing normal saline solution and held at room temperature for 12 to 24 hours [16–18]. The corresponding well numbers were reflected on the patient form. When the plate was full, it was sealed with a transparent adhesive tape. After 12–24 hours, the snips were removed and the fluid from each well was examined separately on a slide for microfilaria under high power (40x) magnification. The results were expressed for each individual as “positive” or “negative” and were recorded in the registration form. Microfilaria prevalence was expressed as a percentage of the number examined [19]. Consent was obtained from individual adults assessed or from parents of the children assessed. Individuals had the option to opt out without fear of repercussions.

*Nodule Survey.* A total of 332 adults of 20 years of age and above who had lived in the area for at least 10 years were examined for nodules from the same communities that were assessed for mf. Every participant was examined in a well-lit private room. Trained health workers performed a palpation examination on the partially undressed participant, paying attention to bony prominences of the torso, iliac crests, and upper trochanter of the femurs. Onchocercal nodules were identified clinically as being firm, painless, and mobile [19–21]. Results were recorded on the form as “positive” or “negative.” Nodule prevalence was expressed as a percentage of the total number of persons examined.

#### 2.3.2. Entomological Survey 

Fly collection was carried out in Bafang and Foumbot for a period of one month. Potential fly collectors of at least 20 years of age were fully informed of the nature of work and the possibility of opting out of the study if they wished so, at any time, without any repercussions. The collectors worked 2 days in Bafang and 15 days in Foumbot in May 1996 near the river banks, where they exposed their legs in shifts from 0600 to 1200 and from 1200 to 1800 hours [19]. As female *Simulium *flies seeking a blood meal settled on the exposed legs, suction tubes were used to catch them before they bit. Using a dissecting microscope (40x magnification), an experienced dissector opened the vector flies' abdomens, thoraxes, and heads. Dissected flies were then examined under a light microscope in order to identify the presence of infection and to count the number of larval stages (L1, L2, and L3) when present. Since fly collection lasted one month, it was not possible to determine the annual transmission potential (ATP). Infective flies were defined as flies with L3s in the head [22, 23].

Monthly biting rate (MBR) was calculated as per the standard method as

(1) MBR=(Number  of  flies  collected×number  of  days  in  the  month)×(Number  of  fly  collection  days)−1.

### 2.4. Follow-Up Assessments

#### 2.4.1. Parasitological Assessments 2005, 2006, and 2011 

Baseline mf rates in the sentinel communities were followed up in only 9 sentinel communities in 2005, three months after mass treatment with ivermectin, in 2006, six months after, and eleven months after treatment in 2011. Three baseline sentinel communities (Foundjanti in Bafang, Bapi in Baham, and Bakassa in Penka Michel) were not assessed in 2005 as they were inaccessible as a result of heavy rains, and the status quo was maintained in 2006. A total of 878 and 780 resident adults were assessed in 2005 for mf and nodules, respectively. Also, 403 resident children (≤10 years old) were assessed for mf.

In 2006, assessment for mf and nodules covered 782 resident adults. Skin snips were obtained from only 134 children. Skin snipping performed in 2005 and 2006 involved two skin snips, one taken from the posterior iliac crest and another from the buttock with the help of a disposable sterile dermal hook and a blade. The hook and blade used for each participant were safely discarded [16].

In 2011, 2703 resident adults from 16 communities, including 11 baseline communities, were examined for mf and nodules. Since the Ministry of Health wanted to know the situation inside and outside the sentinel communities, four additional high risk communities were considered in 2011 assessments. Also 626 resident children (≤10 years) from 11 communities (including three baseline communities) were examined for mf. mf prevalence was expressed as a percentage of the number examined. Due to heavy rains, Bakambe, one of the original sentinel communities, was inaccessible and therefore was not assessed. Nodule palpation was not followed up in the present study as it had not been done in children at baseline.

The comparison of baseline with follow-up results in 2005 (three months after treatment), in 2006 (six months after treatment), and in 2011 (11 months after treatment) was done in order to shed light on the dynamics of *Onchocerca volvulus* infection with annual mass treatment. The comparison of baseline results to follow-up assessments was possible as only qualitative (presence or absence of mf or nodules) data was considered.

#### 2.4.2. Follow-Up Entomological Assessment

Black fly collection was carried out at three sites, in Bafang, Foumbot, and Massangam health districts for 3 days, during the third week of each month, from March 2011 to February 2012. The criteria for selection of potential fly collectors set during baseline entomological assessment were followed. Bafang collection site is located in extreme west of the region Foumbot in the middle and Makouopsap in the extreme east of the region.

Landing female *Simulium* flies were collected and immediately dissected in order to determine the parous rate. The remains of the dissected parous flies were preserved in a tube containing 70% ethanol. The tubes were labeled by collection site, date, and time. *Simulium* flies were then grouped in batches up to 50 and sent to the laboratory where they were stained with Mayer's hematoxylin and fully dissected in search for onchocercal larval stages (L1, L2, and L3) in the abdomen, the thorax, and the head [24]. Infective flies were defined as flies with L3 in the head as in the baseline surveys mentioned previously [18]. This information was used to calculate the monthly and annual transmission potentials which are the indicators of transmission. The annual transmission potential (ATP) was calculated as the sum of the individual monthly transmission potentials (MTPs) over the period of a year [23].

*Data Analysis*. Parasitological data from adults and children as well as entomological data were entered and analysed graphically in Microsoft Excel and Epi Info, CDC, Atlanta GA, USA, for chi square test of independence. The entomological data was analysed and graphically illustrated in Microsoft Excel.

*Ethical Approval*. All the surveys from the baseline to the follow-up studies were approved by the Ministry of Public Health of Cameroon and the National Ethical Committee in Yaoundé. In addition, the Emory University Institutional Review Board (eIRB-11 438) approved and considered them as nonresearch, but routine program evaluation. The followup of 2011 was also conducted under the auspices of World Health Organisation. All assessed individuals had the liberty of opting out of assessments if they wished so without any repercussions.

## 3. Results

### 3.1. Microfilaria (mf) and Nodule Prevalences

**Figure 2:**
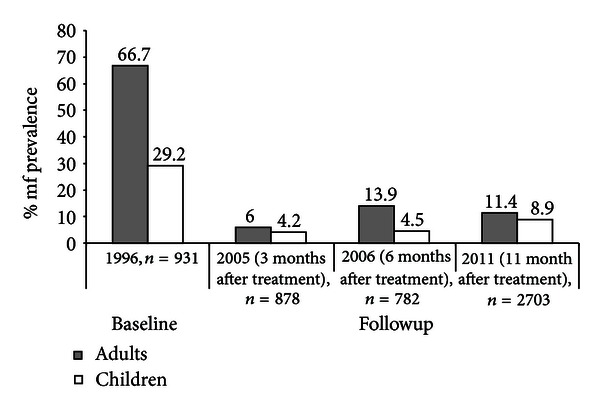
Comparison of mf rates among adults and children at baseline, 1996, with followup surveys in 2005, 2006, and 2011 in West Region of Cameroon.

Among adults, the mf rate reduced by about 91% from baseline level of 66.7% (range 53.1% to 88.1%) in 1996 to 6.0% (range 1.4% to 18.3%, *P* < 0.0001) in 2005, three months after ivermectin treatment. However, mf rate increased in 2006, six months after ivermectin treatment to 13.9% (range 2.1% to 33.6%) although it was not statistically different from 2005 mf rate, *P* < 0.053. The decrease of mf rate, 13.9% in 2006 to 11.4% (range 0% to 59.6%) in 2011, was also not significant (*P* < 0.053) (Table 2 and Figure 2). Only one community had 0% mf rate while six communities had mf rates above 10% and two above 40% after 15 years of annual mass treatment. Persistent high mf rates were observed in communities of Babouantou (21.4%) in Bandja health district, Njone (41.9%) in Foumbot health district, and Makouopsap (59.6%) in Massangam health district. However, there were communities (Bakonti in Bafang Health District, Folap in Foumban, Mbafam in Kekem, and Njisseng in Kouptamo) which registered mf rates below 5% in adults.

In children, overall baseline mf rate of 29.2% (range 12.7% to 51.8%) reduced to 4.2% (range 0% to 25.0%) in 2005 with an 85.6% reduction, *P* < 0.0001 (Table 3 and Figure 2). However, there was no significant difference between mf rate 4.2% in 2005 and 4.5% in 2006, three months and six months respectively after mass treatment. There results for 2006 were also not significantly different from mf rate, 8.9% obtained in 2011, eleven months after treatment (Table 3 and Figure 2). There were children in 2 (13.3%) communities with mf rates above 20% (Njone, 25.2%, and Makouopsap, 65.6%). Even Babouantou with mf rate of 15.8% was considered high for a program with 15 years of annual treatment. In Ndjipta III of Bangangte Health District, Folap of Foumban, and Mbafam of Kekem mf rates among children were 0.6 or less.

The overall baseline nodule rate in adults of 66.3% (range 40% to 89.7%) declined to 9.5% (range 40.0%–89.7%), *P* < 0.0001 in the 2005. This represents a decline of 85.6%. Then, it increased to 18.5% (range 6.7%–30.3%) in 2006 and declined to 12.1% (range 1.5%–43.4%) in 2011 (Table 4). There were 9 communities out of 16 with nodule prevalence of at least 10%. Of particular interest are persistent high nodule rates in Bakambe (23.2%) and Fondjanti (23.2%) communities of Bafang Health District, Fossang-chefferie (17.3%) and Njone (18.6%) in Foumbot health district, and Makouopsap (43.4%) in Massangam Health District.

### 3.2. Entomology

**Figure 3:**
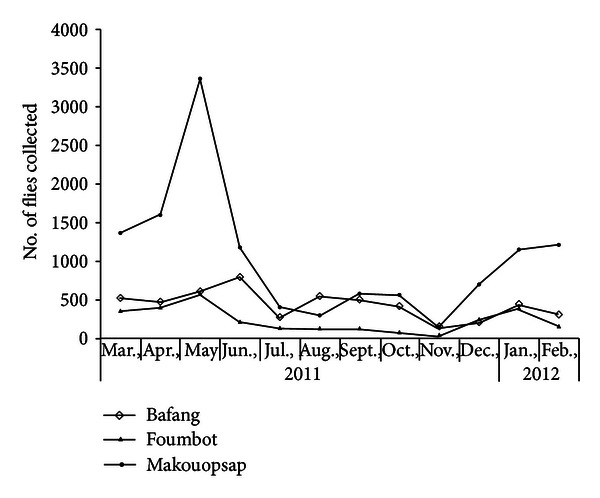
Monthly seasonal biting of *Simulium flies at 3 *fly catching sites in West Region.

Baseline monthly transmission potentials were 15 in Bafang and 210.4 in Foumbot (Table 5). In the follow-up assessment, the infection rates were 0.2 in Bafang Health District, 0.88 in Foumbot, and 0.67 in Massangam. The infective rates were 0 in Bafang Health District, 0.19 in Foumbot, and 0.18 in Massangam. Annual biting rates were 52,610 in Bafang, 28,560 in Foumbot, and 125,360 in Makouopsap, while annual transmission potentials were 0, 70, and 310, respectively. Biting was generally throughout the year, although the main peak biting period in Makouopsap was observed from January to May (Figure 3). 

## 4. Discussion

Annual mass treatment with ivermectin for 15 years had considerably reduced microfilaria and nodule prevalence in all the sentinel communities of West Region of Cameroon. Elimination is considered attained when the microfilaria prevalence in skin snips is less than 5% in sampled communities, in less than 1% in 90% of sampled communities and when entomological criteria of less than 0.5 infected flies/1000 are attained [11]. Among adults, Foumban Health District was close to the epidemiological criterion while Bafang Health District was not very far from the entomological criterion with the ATP of 0. mf rate among children in Foumban and Kekem health districts was zero indicating no recent infection, an indication that interruption of transmission may be attained. However, the mf rates in Baham, Banja, Bangangté, Foumbot, and Massangam health districts among adults and children were still uncomfortably high showing continuing transmission. In adults, nodule rates near or above the threshold 20% for the mass treatment in some communities were of a major concern. The infective rate of 0.18 to 0.19 and ATP of 70 to 300 confirmed continuing transmission.

One possible explanation for high mf rates in children and adults could have been low treatment coverage. However, the methodology for validating UTG treatment coverage followed standard statistical methods for selecting sampled communities and the interviewees. This methodology had been tested and used to validate performance of CDTI in Cameroon and in other onchocerciasis endemic countries [14, 15, 25]. The UTG treatment coverage results were also corroborated by independent monitoring results in unpublished reports supported by APOC. Therefore, there is no reason to believe that UTG treatment coverage was low and responsible for failure to attain optimal parasitological and entomological impacts.

High vector density and mf rates suggest that the force of transmission may have been very high and most likely the reason for the results obtained [26]. The present study did not consider the standard measure of intensity of infection which is related to force of infection, community mf load (CMFL). This requires a calculation that involves weighing the snip and counting the microfilaria which was not done [19]. We recommend that it should be done in future studies.

In Massangam Health District, it is possible that high mf and nodule rates in the follow-up assessments may be due to the “force of infection” across the neighbouring Central Region where peer-reviewed studies indicate considerable onchocerciasis transmission [27, 28]. River Nja, a tributary of River Noun, and River Kichi, a tributary of River Mbam, are known black fly breeding sites responsible for cross-border transmission between West and Central regions. Therefore we recommend collaboration between the regions in order to understand the limits of cross-border onchocerciasis affected area and harmonize intervention if elimination becomes the goal in Cameroon.

Another possible explanation for high mf rates could be related to suboptimal response to ivermectin observed in some onchocerciasis endemic areas of Ghana. The adult female *O. volvulus* worms were resuming microfilaria reproduction more rapidly after ivermectin treatment than would normally be expected, suggesting possible development of resistance to ivermectin [29–31]. We recommend that the possibility of suboptimal response to ivermectin in West Region be investigated.

The microfilaria rate in adults and children tended to follow the expected trend where a single annual dose of ivermectin over a number of years significantly reduced the low mf rates that tend to persist [3]. The observed pattern indicated a tendency for the mf rate to raise a few months after mass treatment until another dose of ivermectin is provided, confirming that microfilarial production is not cumulatively reduced by several annual ivermectin treatments [32]. The mf rate trend at three, six, and eleven months after mass treatment is usually not different from the infection rate within the flies over a period after mass treatment with ivermectin [33]. Ivermectin kills existing microfilariae and tends to exert an “embryostatic effect” by which microfilarial production is suppressed over a few weeks after treatment, but then after, the mf rate begins to increase [32]. Under favourable ecological conditions, interruption of onchocerciasis transmission with annual mass treatment may require many more years before it is attained.

As for twice yearly treatment with ivermectin or when it is coupled with vector control, infection rate continued to fall implying that interruption of transmission could be rapidly attained [1, 33, 34]. We recommend that West Region of Cameroon should consider twice yearly treatment or at least annual treatment with targeted vector control.

In the present study, some communities (Folap and Njisseng) in Foumban and Kouoptamo health districts had mf rates lower than 5% in adults and 0% in children. In these communities, the Diawara et al. criteria are close to being attained, and yet with low levels of infection, transmission is much more efficient than at high levels of infection [35–37]. Thus, if low levels of infection are not detected and controlled, they could result in fast disease recrudescence. Skin snip (microscopy) has low sensitivity of less than 20% at less than 20% nodule rate, and the results obtained may not reflect correct mf endemicity levels [38]. Therefore, interventions in these health districts cannot be halted as disease recrudescence could occur [29, 30]. Where interruption of transmission of onchocerciasis is the objective we recommend a search for affordable, less intrusive, rapid, sensitive, and highly specific diagnostic tools for low level infections in order to validate interruption of onchocerciasis transmission.

The APOC threshold for launching mass treatment is an onchocercal nodule rate of ≥20%. Fondjanti community (Bandja Health District) with nodule rate of 23% and mf rate of 6.4% would pass for mass treatment while Njone community (Foumbot Health District) with nodule rate of 18.6% and mf rate of 41.9% would fail [39]. Nodule rate could also be confounded by the presence of ganglia and *Taenia solium* [40, 41]. The entomological results showed that the risk of contracting onchocerciasis in Foumbot Health District was higher than in Bafang Health District, confirming the reliability of mf rates compared with nodule rates. With the shift from control to elimination of onchocerciasis in Africa, we recommend that nodule prevalence should not be used to determine whether an endemic area should receive mass treatment or not.

Annual biting rates with the range of 28,560 to 125,380 are some of the highest observed globally. Yet infective rate in Bafang from the western part of the region was zero, justifying low mf rates (0.6% in children and a mean of 5.2% in adults). The question would be whether annual mass treatment could be withdrawn without resulting in disease recrudescence. Existing low level transmission with the high annual biting rate of 52,610 could still result in onchocerciasis recrudescence. It was also evident in this study that one-month baseline entomological data was likely to miss peak biting, transmission pattern of *Simulium *vectors, and the calculation of ATP. Therefore collection of entomological data over several months is required as reflected in the follow-up study. The information on peak biting and transmission patterns could effectively be utilized for ivermectin treatment for maximum impact on transmission especially where the force of transmission is considerably high if elimination of onchocerciasis is the goal. In the follow-up survey, it is only at Bafang fly collection site that the entomological criterion for interruption of transmission was met with an ATP of zero [42].

The present study however did not perform molecular testing in order to determine if the L3 larvae were *O. volvulus* or another (animal) *Onchocerca* species. Based on human mf prevalence in skin and infections in children, we think that there is likelihood that some of the larvae observed in vectors were *O. volvulus*. However, a study conducted in North Region of Cameroon during the 1990s showed that 33% of infective larvae in *S. damnosum* were *O. volvulus*, whereas 65% were *O. ochengi* and 2% were *O. ramachandrini* [43]. It is until such a study is conducted in West Region of Cameroon that we will know the extent to which *O. ochengi* is responsible for a significant proportion of infected flies that could confound the infection rate there.

Our findings reflecting an observation period of 15 years showed that annual mass treatment with ivermectin may not interrupt the transmission of onchocerciasis in all different ecological zones of West Region. Therefore, the intensive use of ivermectin is recommended if interruption of transmission of onchocerciasis is to be attained [1].

## 5. Conclusion

Annual mass treatment with ivermectin through community-directed treatment was preferred as a good and less expensive method for controlling onchocerciasis in endemic African countries with assistance from the African Programme for Onchocerciasis Control. The studies in Mali, Senegal, and Nigeria have shown that an annual dose of ivermectin had interrupted transmission of the disease, and all interventions could be halted without the risk of disease recrudescence. However, an annual dose of ivermectin has not interrupted transmission after 15 years of mass treatment in some areas in West Region of Cameroon, just like in North region [8]. It has also been less effective in some onchocerciasis endemic areas in Ghana. The present paper again highlights the fact that for interruption of onchocerciasis transmission, feasible and different but complementary strategic options should be adopted as elimination becomes the goal in Africa.

## Figures and Tables

**Table 1:** Comparing reported and validated (through surveys) UTG treatment coverage in West Region from 2003 to 2010.

Year	2003	2004	2005	2006	2007	2008	2009	2010
Reported coverage	102.6	94.4	91.2	97.6	98.3	95.8	98.5	98.3
Verified through surveys	93.2	95.9	96.7	98.6	90.2	91.4	88.2	83.5
	(*n* = 2370)	(*n* = 2370)	(*n* = 2305)	(*n* = 2436)	(*n* = 2453)	(*n* = 694)	(*n* = 713)	(*n* = 506)

**Table 2:** Comparing mf prevalence among adults at baseline (1996) and followup in 2005, 2006, and 2011, three months, six months, and eleven months, respectively, after mass treatment, in West Region, Cameroon.

District	Community	Baseline, 1996 (*n* = 931)			Followup, 2005 (*n* = 878)			Followup, 2006 (*n* = 782)			Followup, 2011 (*n* = 2703)		
		No. examined	No. positive	% mf positive	No. examined	No. positive	% mf positive	No. examined	No. positive	% mf positive	No. examined	No. positive	% mf positive
	Bakassa	61	36	59.0	140	2	1.4*	97	2	2.1	251	14	**5.6**
Bafang	Bakonti	52	36	69.2	99	3	3.0*	75	3	4.0	338	12	**3.6**
	Fondjanti	124	87	**70.2**							125	8	**6.4**
	Bakambe	122	65	53.3	105	3	2.9*	91	6	6.6	ND	ND	ND

Baham	Bapi	145	77	53.1	ND	ND	ND	ND	ND	ND	189	21	**11.1**

Bandja	Babouantou (Batoula)	68	49	72.1	53	7	13.2*	76	14	18.4	84	18	**21.4**

Bangangté	Batchingou	84	61	72.6	102	11	10.8*	80	14	17.5	247	45	**18.2**
	Ndjipta III (Fop-Tchui)	88	71	80.7	78	1	1.3*	57	6	10.5**	92	9	**9.8**

Foumbot	Fossang-chefferie*	32	28	87.5	71	13	18.3*	72	19	26.4**	150	19	12.7***
	Njone	59	52	88.1	135	11	8.1*	122	41	33.6**	167	70	41.9***

Kekem	Mbafam	39	27	69.2	95	2	2.1*	112	4	3.6	163	8	**4.9**
Penka-Michel	Bakassa	57	32	56.1	ND	ND	ND	ND	ND	ND	195	7	**3.6**
Foumban	Folap	ND	ND	ND	ND	ND	ND	ND	ND	ND	265	0	**0.0**
Kouoptamo	Njisseng	ND	ND	ND	ND	ND	ND	ND	ND	ND	168	5	**3.0**
Malantouen	Matoupou	ND	ND	ND	ND	ND	ND	ND	ND	ND	170	13	**7.6**
Massangam	Makouopsap	ND	ND	ND	ND	ND	ND	ND	ND	ND	99	59	**59.6**

		**931**	**621**	**66.7**	**878**	**53**	6.0*	**782**	**109**	**13.9**	**2703**	**308**	**11.4**

ND: not done.

*Significant (*P* < 0.05)—followup 2005 compared with the baseline.

**Significant (*P* < 0.05)—followup 2006 compared with 2005 followup.

***Significant (*P* < 0.05)—followup 2011 compared with 2006 followup.

**Table 3:** Comparing mf prevalence among children at baseline (1996) and followup in 2005, 2006, and 2011, three months, six months, and eleven months, respectively, after mass treatment, in West Region, Cameroon.

District	Community	Baseline, 1996 (*n* = 185)			Followup, 2005 (*n* = 403)			Followup, 2006 (*n* = 134)			Followup, 2011 (*n* = 626)		
		No. Examined	No. positive	% mf positive	No. Examined	No. positive	% mf positive	No. Examined	No. positive	% mf positive	No. exam	No. positive	% mf positive
Bafang	Bakonti-Bakassa	102	13	12.7	74	0	0*	43	0	0.0	167	1	0.6
Bafang	Batchieu				70	4	5.7	4	0	0.0			
Baham	Bapi	ND	ND	ND							29	2	6.9
Bandja	Babouantou (Batoula)*	ND	ND	ND	64	7	10.9	14	3	21.4	19	3	15.8
Bangangte	Batchingou*	ND	ND	ND	24	1	4.2	15	2	13.3	21	1	4.8
Bangangte	Ndjipta III (Fop-Tchui)*	ND	ND	ND	63	2	3.2	5	0	0.0	25	0	0.0
	Fossang Chefferie	ND	ND	ND	38	0	0.0	4	0	0.0	25	2	8.0
Foumbot	Njone	20	19	95.0	12	3	25*	16	1	6.3	82	24	29.3***
Foumbot	Kousang-Malanden	63	22	34.9	ND	ND	ND	ND	ND	ND	ND	ND	ND
Kekem	Mbafam*	ND	ND	ND	58	0	0.0	33	0	0.0	20	0	0.0
Penka-Michel	Bakassa	ND	ND	ND	ND	ND	ND	ND	ND	ND	74	1	1.4
Foumban	Folap	ND	ND	ND	ND	ND	ND	ND	ND	ND	108	0	0.0
Kouoptamo	Njisseng	ND	ND	ND	ND	ND	ND	ND	ND	ND	24	1	4.2
Massangam	Makouopsap	ND	ND	ND	ND	ND	ND	ND	ND	ND	32	21	65.6

	**12**	**185**	**54**	**29.2**	**403**	**17**	4.2*	**134**	**6**	**4.5**	**626**	**56**	**8.9**

ND: not done.

*Significant (*P* < 0.05)—followup 2005 compared with the baseline.

**Significant (*P* < 0.05)—followup 2006 compared with 2005 followup.

***Significant (*P* < 0.05)—followup 2011 compared with 2006 followup.

**Table 4:** Comparing nodule prevalence among adults at baseline (1996) and followup in 2005, 2006, and 2011, three months, six months, and eleven months, respectively, after mass treatment, in West Region, Cameroon.

District	Community	Baseline, 1996 (*n* = 305)			Followup, 2005 (*n* = 780)			Followup, 2006 (*n* = 782)			Followup, 2011 (*n* = 2703)		
		No. examined	No. positive	% nodule positive	No. examined	No. positive	% nodule positive	No. examined	No. positive	% nodule positive	No. examined	No. positive	% nodule positive
Bafang	Bakassa	27	16	59.3	139	11.0	7.9*	97	11	11.3	251	21	8.4
	Bakonti	27	11	40.7	98	5.0	5.1*	75	5	6.7	338	36	10.7
	Fondjanti	27	19	70.4							125	29	23.2
	Bakambe	27	19	70.4	105	11.0	10.5*	91	13	14.3	125	29	23.2
Baham	Bapi	29	15	51.7	ND	ND	ND	ND	ND	ND	189	15	7.9
Bandja	Babouantou (Batoula)	26	16	61.5	61	5.0	8.2*	76	18	23.7**	84	12	14.3
Bangangté	Batchingou	29	22	75.9	101	16.0	15.8*	80	23	28.8**	247	29	11.7***
	Ndjipta III (Fop-Tchui)	29	23	79.3	78	5.0	6.4*	57	8	14.0**	92	9	9.8
Foumbot	Fossang-chefferie	28	24	85.7	71	7.0	9.9*	72	18	25.0**	150	26	17.3***
	Njone	29	26	89.7	34	9.0	26.5*	122	37	30.3	167	31	18.6***
Kekem	Mbafam	24	17	70.8	93	5.0	5.4*	112	12	10.7**	163	27	16.6
Penka-Michel	Bakassa	30	12	40.0	ND	ND	ND	ND	ND	ND	195	15	7.7
Foumban	Folap	ND	ND	ND	ND	ND	ND	ND	ND	ND	265	4	1.5
Kouoptamo	Njisseng	ND	ND	ND	ND	ND	ND	ND	ND	ND	168	6	3.6
Malantouen	Matoupou	ND	ND	ND	ND	ND	ND	ND	ND	ND	170	9	5.3
Massangam	Makouopsap	ND	ND	ND	ND	ND	ND	ND	ND	ND	99	43	43.4

		**332**	**220**	**66.3**	**780**	**74**	9.5*	**782**	**145**	18.5**	**2828**	**341**	12.1***

ND: not done.

*Significant (*P* < 0.05)—followup 2005 compared with the baseline.

**Significant (*P* < 0.05)—followup 2006 compared with 2005 followup.

***Significant (*P* < 0.05)—followup 2011 compared with 2006 followup.

**Table 5:** Comparing baseline entomological data of 1996 at two fly collection sites and three during 2011.

Month/year of black fly collection	Baseline May 1996		Followup, 2011		
Black fly collection sites	Bafang (Basseu)	Foumbot (Maka)	Bafang	Foumbot	Massangam
Number of *Simulium* caught	5	166	5261	2856	12538
Number of *Simulium* dissected	5	142	5261	2856	12138
Number of parous flies	1	97	1502	1028	2845
Parous rate (%)	20	68.3	28.5	36	23.4
Number of *Simulium* flies infected (L1, L2, L3]	1	12	3	9	19
Infection rate (%)	20.00	8.45	0.20	0.88	0.67
Number of *Simulium* flies infective L3 larval stage in the head	20	2	0	2	5
Infective rate (%)	20.0	2.11	0.00	0.19	0.18
Monthly biting rate per person	75.0	1660.00	na	na	na
Monthly transmission potential*	15.0	210.40	na	na	na
Annual biting rate per person	Na	na	52,610	28,560	125,380
Annual transmission potential	Na	na	0	70	310
